# Dermatitis Due to Paederus Colombinus: Report of an Epidemic Outbreak of 68 Cases in the Province of Darien, Panama

**DOI:** 10.7759/cureus.1158

**Published:** 2017-04-12

**Authors:** Lorenzo Cáceres, Jose A Suarez, Carmela Jackman, Amanda Galbster, Roberto Miranda, Ingrid Murgas, Juan Pascale, Nestor Sosa, Alfonso J. Rodriguez-Morales

**Affiliations:** 1 Entomology Section, Instituto Conmemorativo Gorgas De Estudios De La Salud, Panama City, Panama; 2 Clinical Research Department, Instituto Conmemorativo Gorgas De Estudios De La Salud, Panama City, Panama; 3 Epidemiology, Ministerio De Salud (Minsa), Darién, Panama; 4 Public Health and Infection Research Group, Faculty of Health Sciences, Universidad Tecnológica De Pereira, Pereira, Risaralda, Colombia

**Keywords:** dermatitis linearis, paederus, arthropod, vesicating, dermatitis, panama

## Abstract

**Introduction:**

Contact dermatitis due to *Paederus *is a particular form of accident by animal contact. It is characterized by the sudden onset of erythematous and vesicular lesions with burning sensation on exposed areas of the body. The aim of this study was to describe the epidemiological and clinical findings of an outbreak of *Paederus* dermatitis in Panama.

**Methods:**

Clinical and epidemiological findings of an outbreak of contact dermatitis caused by *Paederus *sp. in the province of Darien in eastern Panama is reported. After reviewing the clinical records, a clinical-epidemiological questionnaire was developed and used in 20 communities where reported cases were found. We captured and collected the specimens for species characterization for three consecutive days using three different methods of capture.

**Results:**

During May-July, 2014, 68 cases of *Paederus* irritant contact dermatitis occurred in 20 communities of the Darien. Fifty-three percent were females. The age group of zero to five years had the highest number of cases, 15 (22%). The most common clinical presentation was the classical linear dermatitis (58%); 42% of the subjects had mirror image lesions, multiple vesicular-pustular lesions, and crust lesions. Symptoms were most commonly reported as a burning sensation (65%), followed by pruritus in 60%, pain (25%), and fever (nine percent). A total of 81 specimens of *Paederus *were collected, 68% in peridomiciliary areas.

**Conclusions:**

This *Paederus *sp. dermatitis report represents one of the largest outbreak described in Latin America and the diagnosis could be confused with others skin diseases like pyodermitis or other contact dermatitis.

## Introduction

Contact dermatitis due to *Paederus *sp. is a way of accident by animal contact. It is characterized by the sudden onset of erythematous and vesicular lesions with burning sensation on exposed areas of the body. The toxin, paederina, from the endolymph of insects of *Paederus *genus causes the disease. Diagnosis is supported by characteristic lineal or mirror lesion patterns and the sudden onset a burning sensation, accompanied by the epidemiological context of the patient [1-2].

The *Paederus *genus has approximately 622 species, 30 of which have been shown to cause lineal dermatitis or to contain the paederin toxic agent [3-5]. *Paederus *belongs to the Staphyllinadae family, Paederinae subfamily, and is widely distributed throughout the world, except in Antarctica [5].

Accidental contact with the insects causes lesions in humans; however, authors in the past believed erroneously that *Paederus *sp. caused these lesions due to biting or stinging. Once the beetle comes in contact with the individual, they will try to brush the insect off, thereby disseminating the paederina, producing contact dermatitis to varying degrees. The severity of the lesion varies by anatomic area. Eye damage such as conjunctivitis and keratitis may occur [4, 6-7].

It is important to note that the clinical diagnosis is eminently epidemiological. In doubtful cases, pathologic examination can be performed to confirm diagnosis; however, findings such as spongiosis and exocytosis of neutrophils, intraepidermal vesicles and epidermal necrosis with dermal edema with interstitial inflammatory infiltrate and perivascular without immunocomplex deposits, should be correlated with the findings of the medical history of patients [6, 8].

Conventional management of contact dermatitis is recommended: removal of irritant, wash with soap and water, and application of moist bandages. Antibiotics are only used if a secondary infection is present. Oral antihistamines and topical corticosteroids are useful in relieving symptoms.

Most outbreaks of *Paederus *dermatitis have been reported in Asia. In the Americas, only 10 reports are available, the last in 2013 and only one of them is from Panama in 1982.

## Materials and methods

The aim of this study is to perform a clinical and epidemiological description of an outbreak of contact dermatitis caused by *Paederus* sp. in the province of Darien in eastern Panama by analyzing the possible causes of this outbreak and to make suggestions for future prevention and control.

In July 2014, an unusually high number of dermatitis cases were observed. Local interpretation was of impetigo or pyoderma, characterized by poor response to antibiotic treatment. This led the regional epidemiologist from the Ministry of Health to investigate the cases.

The first 10-suspected cases were investigated. Evaluation of clinical and epidemiological findings was undertaken. Case definition of the dermatitis lesions was developed for the suspected outbreak of lineal dermatitis due to *Paederus* sp.

Case definition: all skin lesions, characterized as flat and linear with surrounding erythema; one of the following central characteristics: lesion like a burn with crusty appearance or a vesicular lesion with or without history or contact with insect in patients that live in or have visited Darien since May 2014.

Based on the case definition, a retrospective study was performed during the previous two months since the first cases presented. This ensured that individuals who were most likely to be involved in the outbreak were included in this study. The diagnosis at the time of hospital or health center discharge as well as outpatient records to health centers in the towns of, Metetí, Platanilla, Santa Fe were used.

After reviewing the clinical records, a clinical-epidemiological questionnaire was developed and used in 20 communities where reported cases were found. This questionnaire included sociodemographic variables (sex, age, among others), date and place of occurrence, as well clinical manifestations related to the lesions (anatomical location, symptoms, risk factors).

**Figure 1:**
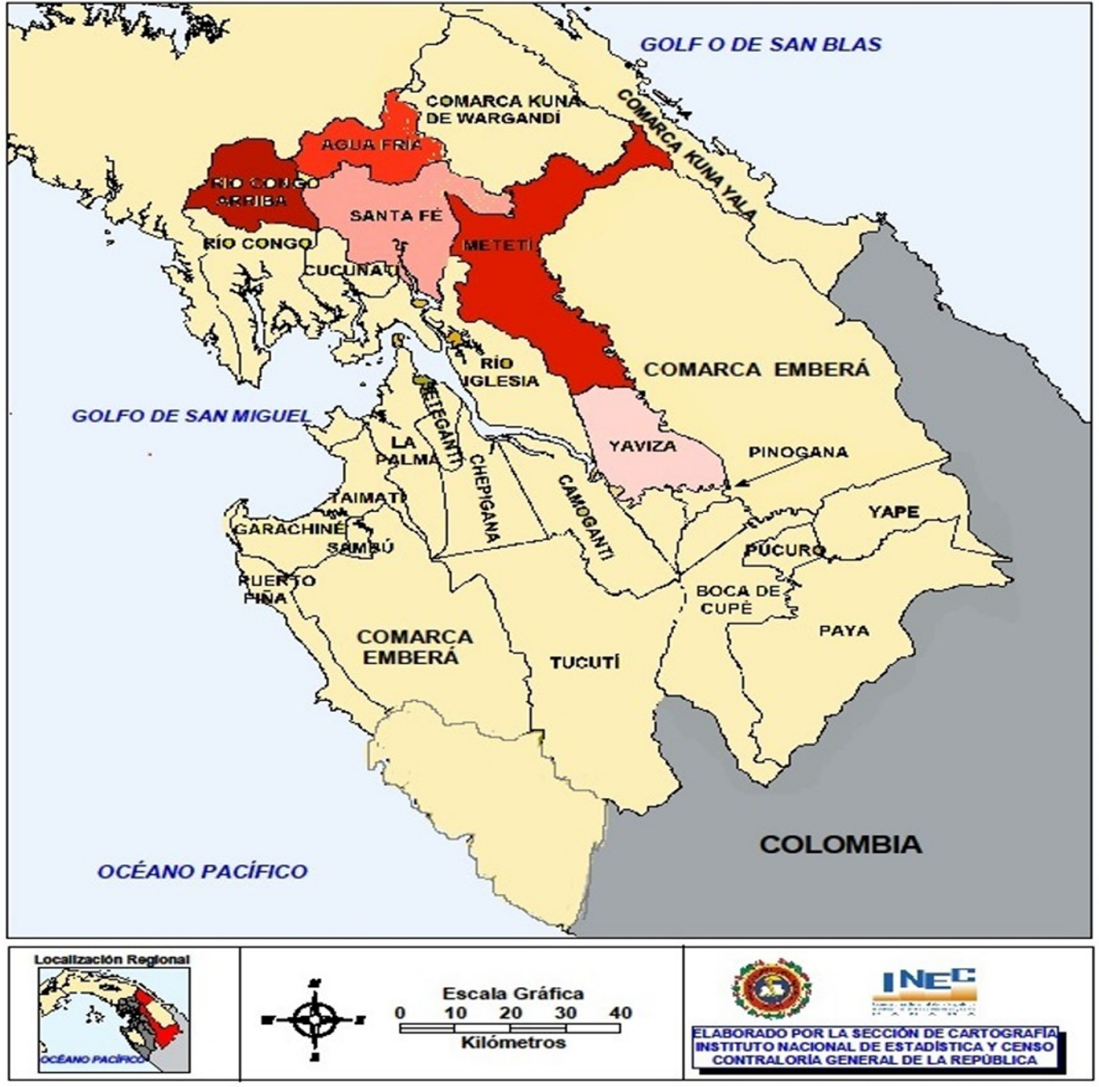
Study area, Darien province, Panama

An epidemiological field study was conducted in the endemic area of *Paederus* sp. This included specimen capture and collection for species characterization. This study was undertaken in districts that reported clinical cases (Rio Congo Arriba, Agua Fria, Santa Fe, Meteti, Yaviza). Three of these districts were chosen (Metetí, Rio Congo Arriba, and Agua Fria) for entomological field search (Figure 1).

Specimens were collected for three consecutive days using three different methods of capture. During the daytime hours, active searching and capture were undertaken using entomological forceps within and around households and selected homes. Nighttime capture using ultraviolet light traps placed around houses was also used. Community members were trained to capture the insects in locations where reported cases were found using containers that were supplied by the study team. All biological material was collected and transferred to previously coded vials containing 70% ethanol and transported to the Department of Medical Entomology at the Gorgas Memorial Institute for identification and classification.

This study has been reviewed and approved by the IRB of Instituto Conmemorativo Gorgas de Estudios de la Salud, Panama City, Panama. In addition to this, informed consent was obtained from the patients, also including that they authorized photographs to be taken and be used in this article.

## Results

**Figure 2:**
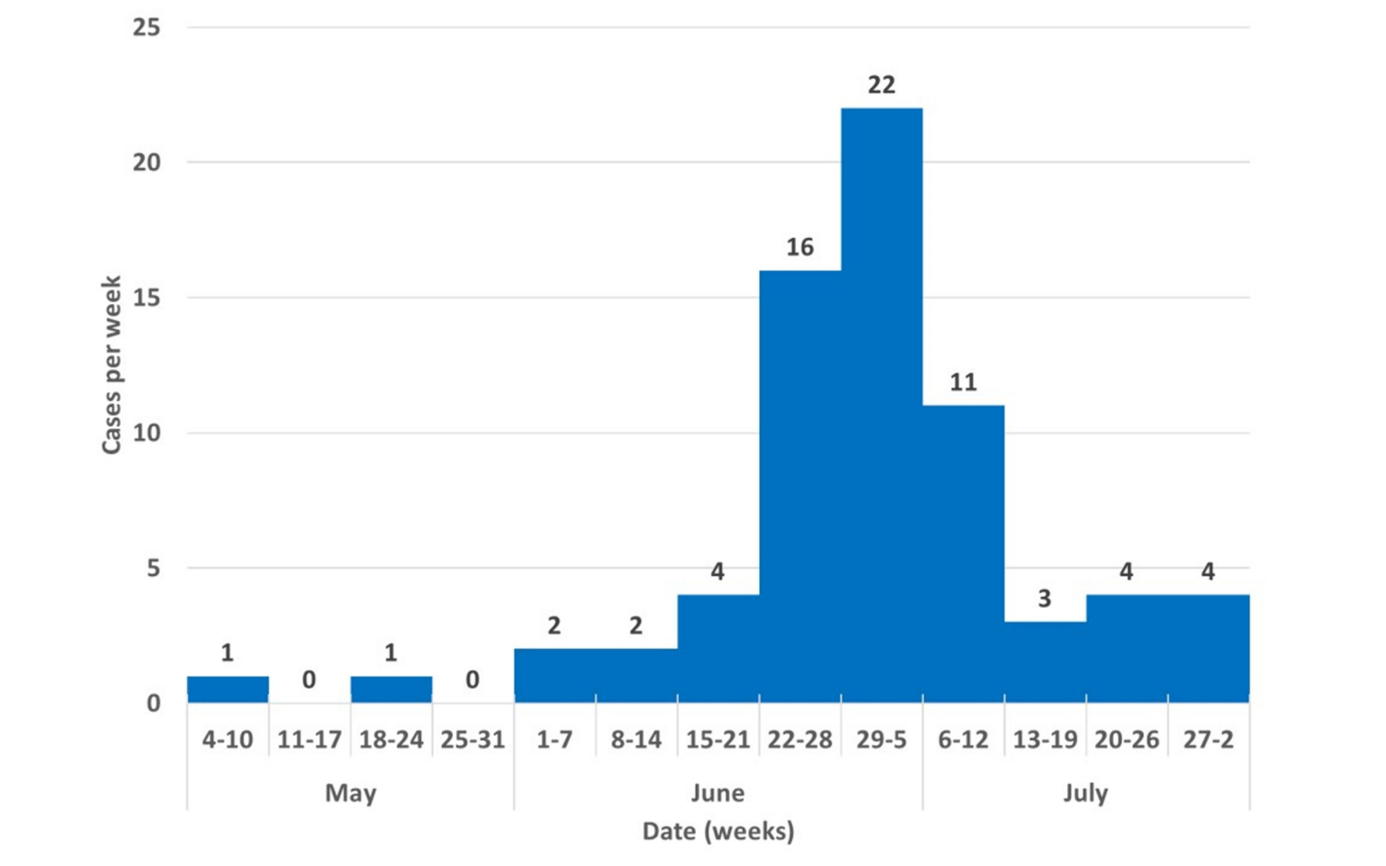
Distribution of Paederus dermatitis cases in Darien, Panama, by epidemiological weeks, May 4-August 2, 2014

During May to July 2014, a total of 68 cases of *Paederus *irritant contact dermatitis were reported in 20 communities of the Darien Province in Panama (Figure 2).

These cases were reported by three different health centers (Platanillas, Santa Fe, and Metetí). The diagnosis was suspected based on the clinical appearance of the lesions.

Fifty-three percent of those affected were females. The age group of 0 to 5 years had the highest absolute number of cases, 15 (22.1%); the highest incidence rate was found in 35-39 year olds, 1.2 per 10,000 (Table 1).

**Table 1:** Distribution by age group and attack rate of contact dermatitis from Paederus, Darien, Panama, 2014 95% CI = 95% confidence interval; y-old = years old.

Variable	N	%	95% CI	Attack rates
Sex				
Male	32	47.05%	35.09-59.45	0.21
Female	36	52.94%	40.55-64.91	0.29
Age (y-old)				
0 – 4.99	15	22.06%	12.90-33.76	1.08
5 – 9.99	7	10.29%	4.24-20.07	0.51
10 – 14.99	6	8.82%	3.31-18.22	0.39
15 – 19.99	2	2.94%	0.36-10.22	0.12
20 – 24.99	10	14.71%	7.28-25.39	0.68
25 – 29.99	6	8.82%	3.31-18.22	0.54
30 – 34.99	3	4.41%	0.92-12.36	0.42
35 – 39.99	7	10.29%	4.2420.07	1.22
40 – 44.99	3	4.41%	0.92-12.36	0.50
45 – 49.99	1	1.47%	0.04-7.92	0.21
50 – 54.99	3	4.41%	0.92-12.36	0.72
55 – 59.99	2	2.94%	0.36-10.22	0.58
60 and older	3	4.41%	0.92-12.36	1.03

A structured interview ordered by the Ministry of Health was applied to 43 of the 68 subjects to better characterize this outbreak as part of an epidemiological investigation. Of those interviewed, 51% were females; the median age was 18 years.

**Figure 3:**
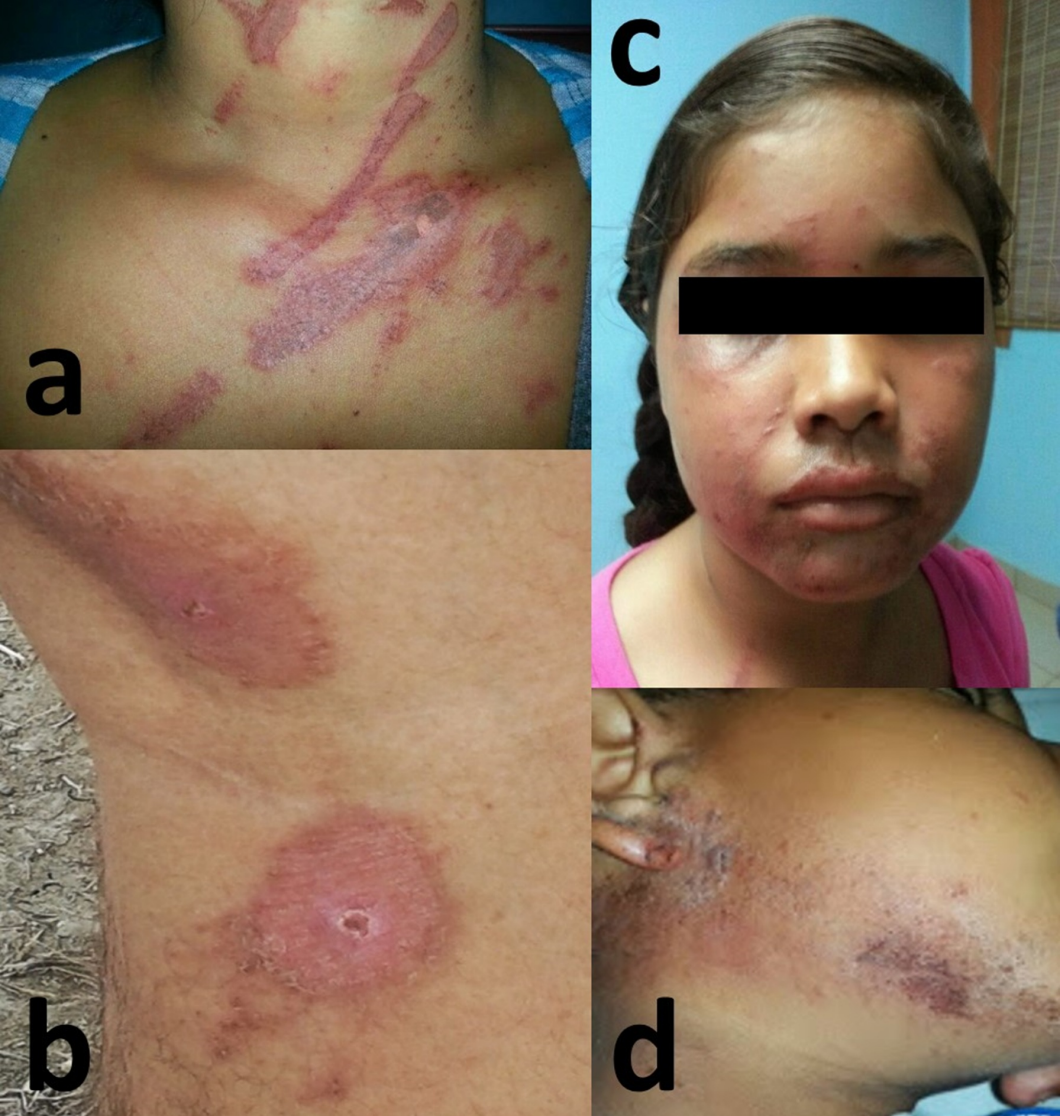
Cutaneous manifestations of dermatitis from Paederus observed during the outbreak. (a) Vesicular linear dermatitis. (b) Mirror lesion. (c) Multiple vesicular-pustular lesions and crust lesions in the face. (d) Multiple vesicular-pustular lesions and crust lesions in the periauricular and mandibular areas

The most common clinical presentation from 43 of the 68 subjects interviewed was the classical linear dermatitis (58.1%). 41.9% of the subjects had mirror image lesions, multiple vesicular-pustular lesions, and crust lesions (Figure 3).

In 17 of 43 subjects, the lesions were in the face, making this the most common anatomical site (39.53%) (Figure 3C). The anatomical site next most affected was arms and lower extremities, this occurred in 27.9% of the patients.

Symptoms were most commonly reported as a burning sensation in 65.1% (28/43), followed by pruritus in 60.4% (26/43); 25% reported pain. Only four patients (9.3%) reported fever.

All individuals interviewed lived in rural communities. The majority, 73% (30/41), did not have metallic screens on the doors or windows. Almost all, 95% (41/43), reported the use of white lights inside and outside of the houses. Ornamental plants and/or fruit trees surrounded most of these houses.

**Figure 4:**
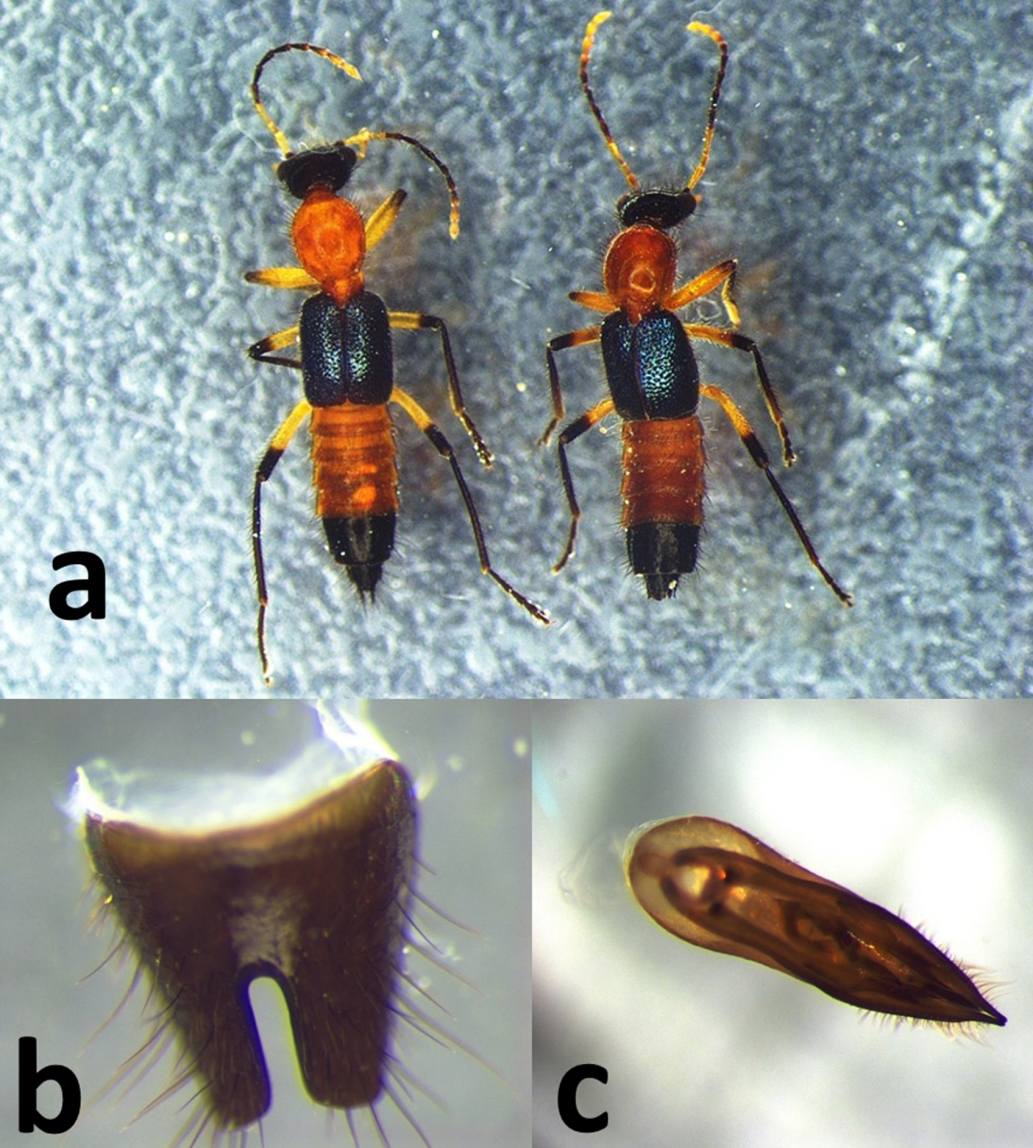
Entomological findings and morphological characteristics for genus identification. (a) Male and female of Paederus captured in Darien. (b) labrum. (c) reproductive male structures.

Regarding the entomological captures, with the three methods used during the three days of fieldwork, we collected 81 specimens of *Paederus* sp. (Figure 4).

The greatest number, 55 (68.0%), of *Paederus* sp. were captured in the areas around houses. More than half, 43 (53.1%), of the samples were found through active searching methods and the remainder was through community participation. Nighttime collection with ultraviolet light traps was not able to capture any *Paederus* sp. specimens.

**Figure 5:**
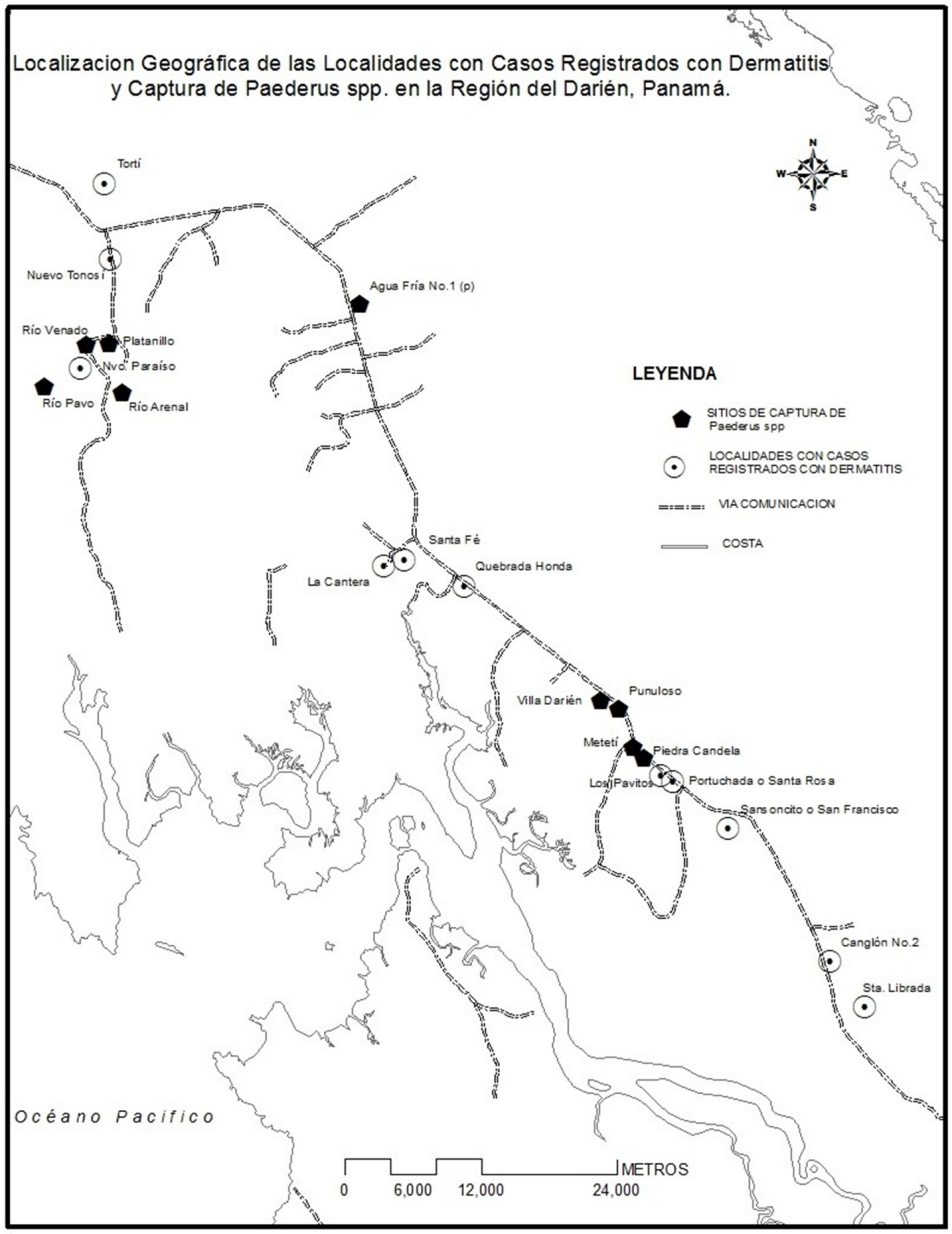
Localization of places with cases of Paederus dermatitis and entomological captures, Darien, Panama

It is important to note that captures were made in four of the five townships where cases of dermatitis were evaluated (Rio Congo, Meteti, Santa Fe, Agua Fria). In the district of Yaviza there were no captures (Figure 5).

The greatest number, 46 (57.0%), of *Paederus* sp. insects were captured in Río Congo Arriba, followed by Metetí with 31 (38.3%) of the captures, Santa Fe and Agua Fria, each with two (2.5%) captures (Table 2) (Figure 5).

**Table 2:** Captures of Paederus spp. (Coleoptera: Staphylinidae), in places were dermatitis cases occurred, Darien, Panama

Corregiment	Localization	N	%, capture	Place	Type of collection
Río Congo Arriba	Platanillas	8	9.9	Peridomestic	At community
Río Congo Arriba	Río Venado	5	6.2	Intradomicilio	Active search
Río Congo Arriba	Río Arenal	28	35	Peridomestic	Active search
Río Congo Arriba	Agua Fría	5	6.2	Peridomestic	At community
Meteti	Punuloso	9	11.1	Intradomicilio	At community
Meteti	Meteti	8	9.9	Intradomicilio	At community
Meteti	Piedra Candela	6	7.4	Peridomestic	At community
Meteti	Villa Darién	8	9.9	Peridomestic	Active search
Santa Fe	Santa Fe	2	2.5	Intradomicilio	At community
Agua Fría	Agua Fría No. 1	2	2.5	Intradomicilio	Active search

## Discussion

The *Paederus *sp. releases a toxic hemolymph (Paederina) which generally occurs when the beetle is brushed or rubbed on the skin or when there is accidental contact with the skin. An inflammatory reaction, known as contact dermatitis from *Paederus* sp. or lineal vesicular dermatitis occurs [9-10]. In Panama, the species *P. signaticornis *and *P. columbinus *have been reported to cause severe dermatitis. In Central America, the species *P. laetus, P. ardus, P. luridiventris*,and *P. salivini *exist, however only *P. laetus *has been shown to cause cases of dermatitis in Guatemala [11-12].

The outbreak of dermatitis in the Darien region is characterized by an unusual increase in the population of *Paederus *sp. as well as several cases of dermatitis in villages that are located adjacent to the Pan American highway. Most the cases ranged along 110 km of highway from the western extreme at the town of Tortí, located at the eastern part of the province of Panama, to the most eastern town of Yaviza in the province of Darien. The increase in the population of the *Paederus* sp. was found with an invasion of these beetles in the houses. The insects most likely were attracted by the light which brought them into the rooms where they met humans. The patient reports show that dermatitis by *Paederus* sp. occurred mostly at night. This species of beetle is characterized by having mostly nocturnal activity and are attracted by artificial light [11-12].

In Pakistan, a *Paederus *sp. dermatitis outbreak showed most of the affected persons worked at night near artificial lights; the beetles were collected directly below light sources [13]. This outbreak involved 191 cases [13]. It is common for the insects to become active an hour after sunset [14]. The species showed variable behavior, which indicates their attraction to ultraviolet and white light; they are relatively insensitive to orange and yellow light [15]. However, in an outbreak in Tanzania, ultraviolet light was not found to attract the insects but the incandescent yellow light did [16].

Collection sites for *Paederus *sp. in this study were natural ecological areas, including savannah or grasslands with varying amounts of surrounding shrubs and secondary forests that were invaded by anthropogenic activity. Houses were mostly located near grasslands and secondary forests. Dwellings had favorable conditions in regards to basic health services, such as sewage disposal, water, electricity and general hygiene and sanitation. One of the social and environmental aspects, which could eventually contribute to *Paederus* sp. incidents in the region, is that although almost all homes maintain good hygiene, many people keep the windows open at night without the use of protective screens. This may constitute an important factor for contact with *Paederus *sp. [4].

In Malaysia, *P. fuscipes *was found inside homes, mainly in bedrooms, hallways, in bathrooms, and on walls [12]. Those who live in homes under impoverished conditions may be two times more likely to become ill than the rest of the community. That said, in our investigation, housing conditions were not a differentiating factor [17-18].

The unusual increase in the *Paederus *sp. population and the increased dermatitis cases have been shown to occur during the rainy season [19-20]. Ecological changes and climatic variations, such as El Niño, have also led to the appearance of unusual outbreaks of dermatitis caused by some species of these insects, which increase year after year in some regions worldwide [21-22]. Global warming can also lead to increased incidence of *Paederus *sp. since higher temperatures may influence the ecological dynamics of some insect species [23].

The increases in *Paederus *sp. populations and the dermatitis case reports in this outbreak in the province of Darien could be due to climatic changes related to the onset of the rainy season; unfortunately, due to the limitation of current study, such variables were not available during the outbreak period in order to correlate them with the case incidence. Heavy rainfall and consequently the increase of vegetation in deforested areas and along with the change in temperature and humidity cause an alteration in the natural habitat of these insects. This leads to the migration and proliferation of these insects to more urbanized areas, especially where there is artificial light. The elimination of natural predators has created an ecological imbalance in the habitat of *Paederus *sp. [21-22].

Thermal fogs and residual spraying with pyrethroid insecticides inside and outside dwellings can be used to control insects that invade and remain in houses. In an outbreak of dermatitis *Paederus *sp. in Malaysia, the use of thermal fog, residual spraying, and adhesive material was placed near the light sources to control the insects [24]. Also, other mechanisms in order to prevent the contact with the Paederus – such as not to crush these insects, learn to identify, close window or doors – are important to be instructed in the population.

Epidemic dermatitis by *Paederus *sp. described in South America is related to climatic phenomenon like the El Niño. These outbreaks affected urbanized areas, like what we describe in the Darien outbreak. In an outbreak in Peru [13], adjacent townships were involved, like the outbreak we studied in Darien.

From a clinical point of view, there are two main families of beetles that could be confused during identification. The family meloidea has two genuses, which are Lytta and Epicauta; these produce a powerful irritant known as cantharidin, which is observed in bullous lesions of skin and mucosal tissue. Insects of the family Staphylinidae, which has the *Paederus *genus, cause the classical lineal dermatitis described in this study. The amide discharge has a vesicant greater than cantharidin and causes intense erythema vesicles and scabs in humans [25-30].

Our patients had classical dermatitis lesions from *Paederus *sp., as described in the literature [4]. We observed many cases with facial lesions and lesions on exposed areas. Burning and itching were reported in our group as in other reported cases and outbreaks described in the literature. Systemic symptomatic fever was only reported in a low percentage of cases [26-27].

## Conclusions

This *Paederus* sp. dermatitis report represents one of the largest outbreak described in Latin America and the diagnosis could be confused with other skin diseases like pyodermitis or other contact dermatitis. Then, dermatitis linearis differential diagnosis should be considered in cases with these findings, specially in Panama and other countries of the region.
